# AGE-FRIENDLY LOWELL: INTERGENERATIONAL CONNECTION THROUGH EXPERIENTIAL LEARNING OPPORTUNITIES

**DOI:** 10.1093/geroni/igac059.577

**Published:** 2022-12-20

**Authors:** Michelly Townsend, David Cornell, Andrew Hostetler, Robin Toof, Karen Devereaux Melillo, Sabrina Noel

**Affiliations:** University of Massachusetts Lowell, Wakefield, Massachusetts, United States; University of Massachusetts Lowell, Lowell, Massachusetts, United States; University of Massachusetts Lowell, Lowell, Massachusetts, United States; University of Massachusetts Lowell, Lowell, Massachusetts, United States; University of Massachusetts Lowell, Lowell, Massachusetts, United States; University of Massachusetts Lowell, Lowell, Massachusetts, United States

## Abstract

The rapid growth of the aging demographic requires students entering the workforce to be adept at engaging with older adults in a professional capacity. The Age Friendly Community (AFC) and Age Friendly University (AFU) initiatives present an opportunity for researchers and students to work alongside older residents to improve Age Friendly practices, policies, and infrastructure in their local communities. Age Friendly Lowell developed an Action Group (AG) of 25 Lowell residents aged 50y+, who provide feedback and guidance for 12 graduate students, as they develop measurement tools for a comprehensive community assessment. Students work with AG members to identify, pilot test, and adapt evaluation tools and learn innovative strategies for recruitment. This experiential learning structure is mutually beneficial, as residents lend their expertise, while students learn to echo those concerns throughout their work. This structure serves as a unique and effective model for other communities pursuing AFC and AFU designations.

